# Metabolic Phenotypes in Pancreatic Cancer

**DOI:** 10.1371/journal.pone.0115153

**Published:** 2015-02-26

**Authors:** Min Yu, Quanbo Zhou, Yu Zhou, Zhiqiang Fu, Langping Tan, Xiao Ye, Bing Zeng, Wenchao Gao, Jiajia Zhou, Yimin Liu, Zhihua Li, Ye Lin, Qing Lin, Rufu Chen

**Affiliations:** 1 Department of Hepatobiliary Surgery, Sun Yat-sen Memorial Hospital, Sun Yat-sen University, Guangzhou, Guangdong Province, China; 2 General Surgery Department, Guangdong General Hospital, Guangdong Academy of Medical Sciences, Guangzhou, Guangdong Province, China; 3 Department of Medical Oncology, Sun Yat-sen Memorial Hospital, Sun Yat-sen University, Guangzhou, Guangdong Province, China; 4 Department of General Surgery, Sun Yat-sen Memorial Hospital, Sun Yat-sen University, Guangzhou, Guangdong Province, China; 5 Department of Radiotherapy, Sun Yat-sen Memorial Hospital, Sun Yat-sen University, Guangzhou, Guangdong Province, China; University of South Alabama Mitchell Cancer Institute, UNITED STATES

## Abstract

**Introduction:**

The aim of present study was to profile the glucose-dependent and glutamine- dependent metabolism in pancreatic cancer.

**Methods:**

We performed Immunohistochemical staining of GLUT1, CAIX, BNIP3, p62, LC3, GLUD1, and GOT1. Based on the expression of metabolism-related proteins, the metabolic phenotypes of tumors were classified into two categories, including glucose- and glutamine-dependent metabolism. There were Warburg type, reverse Warburg type, mixed type, and null type in glucose-dependent metabolism, and canonical type, non-canonical type, mixed type, null type in glutamine-dependent metabolism.

**Results:**

Longer overall survival was associated with high expression of BNIP3 in tumor (p = 0.010). Shorter overall survival was associated with high expression of GLUT1 in tumor (P = 0.002) and GOT1 in tumor (p = 0.030). Warburg type of glucose-dependent metabolism had a highest percentage of tumors with nerve infiltration (P = 0.0003), UICC stage (P = 0.0004), and activated autophagic status in tumor (P = 0.0167). Mixed type of glucose-dependent metabolism comprised the highest percentage of tumors with positive marginal status (P<0.0001), lymphatic invasion (P<0.0001), and activated autophagic status in stroma (P = 0.0002). Mixed type and Warburg type had a significant association with shorter overall survival (P = 0.018). Non-canonical type and mixed type of glutamine-dependent metabolism comprised the highest percentage of tumors with vascular invasion (p = 0.0073), highest percentage of activated autophagy in tumors (P = 0.0034). Moreover, these two types of glutamine-dependent metabolism were significantly associated with shorter overall survival (P<0.001). Further analysis suggested that most of tumors were dependent on both glucose- and glutamine-dependent metabolism. After dividing the tumors according to the number of metabolism, we found that the increasing numbers of metabolism subtypes inversely associated with survival outcome.

**Conclusion:**

Warburg type, non-canonical type and mixed types of glucose- and glutamine-dependent metabolism comprised of more metabolically active, biologically aggressive and poor prognostic tumors. Moreover, the increasing subtypes and categories of the metabolism in each tumor significantly associated with poor prognosis.

## Introduction

Aberrant metabolism is now considered as one of the hallmarks of cancer [1]. Notably, tumor cells exhibit high levels of glycolysis even under the adequate oxygen supply, a remarkable phenomenon termed Warburg effect. The pioneering work of Otto Warburg has been supported by reports in various tumor types, and is now exploited in the clinic for diagnostic purposes. However, this view is challenged by emerging evidences. In his theory, Warburg supposed that cancer cells tended to generate ATP through glycolysis instead of oxidative phorsphorylation following the mitochondrial dysfunction [2]. Nevertheless, mounting evidences indicated that primary defects in mitochondrial enzymes or complexes in oxidative phorsphorylation were not frequently observed in cancers [3]. The relatively low number of metabolic inhibitors were introduced in cancer treatment and only a few of them have entered clinical development [4], which partly reflected the heterogeneity and complexity in tumor metabolism. Moreover, study conducted by Zu and his colleague showed that the amount of ATP derived from glycolysis does not exceed 50–60% of total ATP production [5,6]. A new paradigm named “reverse Warburg effect” in which aerobic glycolysis took place in cancer-associated fibroblasts, rather than tumor cells, also emerged in breast cancer [7,8]. Recent data showed that pancreatic cancer cells utilize glutamine in an unusual way, which is dependent on glutamic-oxaloacetic transaminase rather than glutamate dehydrogenase [9]. These findings put a great emphasis on significant heterogeneity in cancer metabolism suggesting that each cancer has its own metabolic features, which needs to be taken into consideration before exploring new targets in cancer metabolism.

Pancreatic cancer is the fourth leading cause of cancer-related deaths in the United States and Europe. It is still one of the most intractable and fatal disease because of its late stage when diagnosed, invasive phenotype, resistant to current therapies [10,11]. Targeting cancer metabolism is a promising strategy in improving pancreatic cancer treatment. To pave the way for development of metabolism therapeutic treatments, metabolism phenotypes in pancreatic cancers are needed to be elucidated. To our best knowledge, there are no studies investigating the metabolic phenotypes in pancreatic cancer. Therefore, the aim of our present study was conducted to profile the glucose-dependent and glutamine-dependent metabolism, mitochondrial, and autophagic status in pancreatic cancer.

## Materials and Methods

### Patients’ selection

Samples of 106 pancreatic cancer tissues were derived from patients undergoing radical operations in a retrospective study cohort between Jan. 2000 and Dec. 2012 at Sun Yat-sen Memorial Hospital of Sun Yat-sen University and a prospective cohort from Jun.2012 to May 2013 (registration number: ChiCTR-TRC-12002548). This study complied with the Helsinki Declaration and was approved by the Institutional Ethics Committee of Sun Yat-sen Memorial Hospital. All patients signed consent forms indicating their willingness to participate and their understanding of the procedure and general aim of the study. All of the included pathological and histological confirmed patients with pancreatic cancer met the following criteria: no history of any other malignant tumor, without neoadjuvant therapy prior to the surgery. None of them died during the perioperative period because of post-operative complications. All patients consent was obtained prior to collection of specimens, and all study procedures were approved by the Ethic Committee Sun Yat-sen Memorial Hospital of Sun Yat-sen University. Data items were extracted through medical record system as following: age, gender, status of glucose metabolism, tumor size, tumor location, histological grade, marginal status, nerve infiltration, vascular invasion, UICC Stage (Tumor pathological stage was classified according to the Union for International Cancer Control (UICC)), and lymphatic invasion. Overall Survival (OS) was defined as the time interval from the date of operation to death or the last follow-up in May 2013.

### Immunohistochemistry

Immunohistochemical analysis was carried out as following: Tissue samples were fixed in neutral-buffered formalin, and then embedded by paraffin. Endogenous peroxidase was quenched with 3% hydrogen peroxide for 30min at room temperature. Slides were then washed in PBS and incubated for 30min at 37°C with trypsin buffer. After adequate wash in PBS, antigen retrieval of 5-μm thick sections of 106 pancreatic cancer tissues was carried out in citrate buffer (PH6.0). When the slides were cooled down to room temperature, nonspecific protein binding were block for 60min by normal goat serum at room temperature. Subsequently, primary antibody (listed in Table 1 in S2 File) for immunohistochemistry was applied overnight at 4°C. Next, Elivision Super DAB kit was applied as manufactory instructions. The primary antibody incubation was omitted as negative controls. Positive controls were chosen as suggested in antibody instructions or previous data [12,13]: glucose transporter 1 (GLUT1): esophageal carcinoma; carbonic anhydrase IX (CAIX): renal carcinoma; BCL2/adenovirus E1B 19-kDa interacting protein 3 (BNIP3), kidney tissue; Beclin-1: breast tissue; microtubule-associated protein 1 light chain 3 (MAPLC3): nerve tissue in each specimens; p62: spleen tissue; glutamic-oxaloacetic transaminase (GOT1): kidney tissue; glutamate dehydrogenase 1 (GLUD1): heart tissue (Figure A in S1 File). Immunoreactivity-scoring (IRS) system was employed for semiquantitative analysis. In brief, the IRS score for each specimen was obtained by two independent pathologists and the mean value of the scores was the final score we used for further analysis. Any Inconsistencies between pathologists’ data were resolved by discussion with a third pathologist until consistency was reached. Scores for expression of metabolism-related protein were evaluated according to intensity and proportion. The intensity score was determined as 0 (no staining), 1 (weaker staining than positive control), 2(equal staining as positive control), and 3 (stronger staining than positive control). The proportion score was determined as 0(negative), 1 (<30% of tumor cells) and 2 (≧30% of tumor cells). The intensity score and proportion score were multiplied together for a total score. Total scores were categorized as follows: 0–1 (negative) and 2–6 (positive).

### Classification of tumor metabolic phenotypes

We classified the cases into phenotypes according to the immunohistochemical staining for these metabolism-related proteins as previous report [12,13]: glycosis types: GLUT1- and/or CAIX-positive; nonglycolysis type: GLUT1- and CAIX-negative; dysfunctional mitochondrial type: BNIP3-positive; functional mitochondrial type: BNIP3-negative; activated autophagy types: two or more autophagic markers from Beclin1, LC3, p62; inactivated autophagy type: positive for less than two autophagic markers from Beclin1, LC3, p62; canonical glutamine metabolism type: GLUD1-positive and GOT1 positive; non-canonical glutamine metabolism type: GLUD1-negative and GOT1 positive. Metabolism in pancreatic cancer was divided into two major categories: glucose-dependent metabolism and glutamine-dependent metabolism [9,14]. Glucose-dependent metabolism phenotype of individual pancreatic cancer specimen is classified as follow according to previous reports [12,13]: Warburg type: tumor (glycolysis type), stroma(non-glycolysis type, oxidative phosphorylation); reverse Warburg type: tumor(non-glycolysis type, oxidative phosphorylation), stroma (glycolysis type); mixed type: tumor (glycolysis type), stroma (glycolysis); and null type: tumor (non-glycolysis type, oxidative phosphorylation), stroma (non-glycolysis type, oxidative phosphorylation). Glutamine-dependent metabolism phenotype of individual pancreatic cancer specimen is classified as follow: canonical type (GLUD1-positive and GOT1-negative), non-canonical type (GOT1-positive and GLUD1-negative), mixed type (GLUD1-positive and GOT1-positive), null type (GLUD1-negative and GOT1-negative) of glutamine metabolism.

### Quantitative Real-time Reverse Transcription-PCR Assay

We assessed the mRNA expression of the target genes with qRT-PCR. Total RNA was isolated from tissues and cell lines using Trizol reagent (Invitrogen), and the concentrations were measured with a NanoDrop ND-1000 spectrophotometer (NanoDrop Technologies). The first strand of the cDNA was synthesized with PrimeScript® 1st Strand cDNA Synthesis Kit (Takara). QRT-PCR was applied according to the manufacturer's instruction. QRT-PCR was then performed at 95°C for 10 minutes, at 95°C for 15 seconds, and at 60°C for 15 seconds, with a total of 40 cycles. Each reaction was performed in triplicate and all the primers used were listed in supplemental data. Primers were designed based on the sequence of each gene by using Premier 5.0. The mRNA levels were normalized to beta-actin mRNA according to the following formula: [2̂ − (C_T_^Target^ − C_T_^Actin^)] × 100%, where C^T^ is the threshold cycle.

### Statistical analyses

Data was analyzed using SPSS for Windows version 16.0 software (SPSS Inc, Chicago, IL, USA). Categorical variables were compared using Fisher exact test. The median survival times were evaluated by Kaplan Meier survival curves, and the differences were further analyzed with the log-rank test. When P value was less than 0.05, significance was assumed unless marked. When P value was less than 0.1 in univariate analysis and these variables were further analyzed in multivariate analysis through cox proportional hazards models.

## Results

### Clinicopathological characteristics of included patients

The present cohort consisted of 106 patients including 57 men and 49 women. The mean age at operation was 60.42 years, with a median age of 61.00 years and a range from 31 years to 77 years. At the time of last clinical follow-up (May 2013), 29 (27.4%) patients were alive. The median overall survival of the 106 patients was 21.9 months, with 1-, 3-, and 5-year survival rates of 56.8%, 13.2%, and 6.2%, respectively. The majority of patients (80.2%) had tumors located at the head of pancreas, and sizes of most tumors (80.2%) were more than 20 mm in greatest diameter. Most tumors were moderately differentiated (37.7%), followed by well differentiated (35.9%), and poorly differentiated (26.4%). Twenty-three (21.7%) patients got a positive margin. Nerve infiltration was presented in 84 patients (79.2%), vascular invasion in 82 (77.4%) patients, and lymphatic invasion in 68 (64.2%) patients. Most patients (73.6%) presented with the Union for International Cancer Control (UICC) stage IIB disease, and the remaining (26.4%) patients presented with UICC stage IA, IB and IIA disease. No patients presented with UICC stage III and IV disease.

### Correlation between the expression of metabolism-related proteins and clinicopathological parameters

The correlation between metabolism-related proteins and clinicopathological parameters were summarized in Table 1 to Table 4. We found that increased tumor expression of GLUT1 was associated with nerve infiltration (P = 0.0180), lymphatic invasion (P<0.0001), UICC stage (P = 0.0044), whereas stromal expression of GLUT1 was associated with tumor dedifferentiation (P = 0.0155), positive marginal status (P = 0.0063), nerve infiltration (P = 0.0005), lymphatic invasion (P = 0.0065) and UICC stage (P = 0.0010) (Table 1). Tumor expression of GLUD1 was associated with larger tumor size (P = 0.0086). Tumor expression of GOT1 was significantly linked with tumor differentiation (P = 0.0238), vascular invasion (P = 0.0384), lymphatic invasion (P = 0.0367). There was a significant association between stromal expression of GOT1 and nerve infiltration (P = 0.0087) (Table 2). Tumor expression of BNIP3 was associated with tumor dedifferentiation (P = 0.0155), whereas stromal expression of BNIP3 was associated with positive marginal status (P = 0.0010) (Table 3). Cytoplasmic p62 expression in tumor was associated with positive marginal status (P = 0.0132), while nuclear p62 expression in stroma was associated with larger tumor size (P = 0.0173) (Table 3). Tumor expression of LC3 was linked with impaired glucose metabolism (P = 0.0205), larger tumor size (P = 0.0453), tumor dedifferentiation (P = 0.0040), vascular invasion (P = 0.0044), lymphatic invasion (P = 0.0385), while stromal expression of LC3 associated with tumor dedifferentiation (P<0.0001), positive marginal status (P = 0.0010), negative vascular invasion (P = 0.0015) (Table 4). There was a significantly association between cytoplasmic expression of Beclin-1 in tumor and lymphatic invasion (P = 0.0175), whereas nuclear Beclin-1 expression in stroma was associated with positive marginal status (P = 0.0379) (Table 4).

**Table 1:** Associations of GLUT1, CAIX expression with clinicopathological parameters *.

Parameters	No. of cases (%)	GLUT1 in tumor			GLUT1 in stroma			CAIX in tumor			CAIX in stroma		
		Negative (n = 44) (%)	Positive (n = 62) (%)	P-value	Negative (n = 75) (%)	Positive (n = 31) (%)	P-value	Negative (n = 82) (%)	Positive (n = 24) (%)	P-value	Negative (n = 95) (%)	Positive (n = 11) (%)	P-value
Age				1.0000			0.5272			0.4905			0.5342
<61 years	50(47.2)	21(47.7)	29(46.8)		37(49.3)	13(41.9)		37(45.1)	13(54.2)		46(48.4)	4(36.4)	
≥61 years	56(52.8)	23(52.3)	33(53.2)		38(50.7)	18(58.1)		45(54.9)	11(45.8)		49(51.6)	7(63.6)	
Gender				0.5568			0.5248			0.8165			1.0000
Male	57(53.8)	22(50.0)	35(56.5)		42(56.0)	15(48.4)		45(54.9)	12(50.0)		51(53.7)	6(54.5)	
Female	49(46.2)	22(50.0)	27(43.5)		33(44.0)	16(51.6)		37(45.1)	12(50.0)		44(46.3)	5(45.5)	
Diabetes mellitus or hyperglycemia				0.3048			0.2578			0.6269			1.0000
No	35(33.0)	12(27.3)	23(37.1)		22(29.3)	13(41.9)		26(31.7)	9(37.5)		31(32.6)	4(36.4)	
Yes	71(67.0)	32(72.7)	39(62.9)		53(70.7)	18(58.1)		56(68.3)	15(62.5)		64(67.4)	7(63.6)	
Tumor Size				0.8887			0.0590			0.2439			0.4526
≤2.0cm	21(19.8)	9(20.5)	12(19.4)		11(14.7)	10(32.3)		14(17.1)	7(29.2)		18(18.9)	3(27.3)	
>2.0cm	85(80.2)	35(79.5)	50(80.6)		64(85.3)	21(67.7)		68(82.9)	17(70.8)		77(81.1)	8(72.7)	
Tumor Location				0.6228			0.4217			0.0588			0.4526
Head	85(80.2)	34(77.3)	51(82.3)		62(82.7)	23(74.2)		13(15.9)	8(33.3)		77(81.1)	8(72.7)	
Body/tail	21(19.8)	10(22.7)	11(19.7)		13(17.3)	8(25.8)		69(84.1)	16(66.7)		18(18.9)	3(27.3)	
Differentiation				0.8254			0.0155			0.9765			0.9948
Well	38(35.9)	15(34.1)	23(37.1)		31(41.3)	7(22.6)		29(35.4)	9(37.5)		34(35.8)	4(36.4)	
Moderate	40(37.7)	16(36.4)	24(38.7)		30(40.0)	10(32.2)		31(37.8)	9(37.5)		36(37.9)	4(36.4)	
Poor	28(26.4)	13(29.5)	15(24.2)		14(18.7)	14(45.2)		22(26.8)	6(25.0)		25(26.3)	3(27.2)	
Marginal status				0.0898			0.0063			0.1159			0.2483
R0	83(78.3)	38(86.4)	45(72.6)		64(85.3)	19(61.3)		67(81.7)	16(66.7)		76(80.0)	7(63.6)	
R1	23(21.7)	6(13.6)	17(27.4)		11(14.7)	12(38.7)		15(18.3)	8(33.3)		19(20.0)	4(36.4)	
Nerve Infiltration				0.0180			0.0005			0.0941			1.0000
Negative	22(20.8)	14(31.8)	8(12.9)		9(12.0)	13(41.9)		14(17.1)	8(33.3)		20(21.1)	2(18.2)	
Positive	84(79.2)	30(68.2)	54(87.1)		66(88.0)	18(58.1)		68(82.9)	16(66.7)		75(78.9)	9(81.8)	
Vascular invasion				0.8144			0.3121			0.0933			0.7091
No	24(22.6)	9(20.5)	15(24.2)		15(20.0)	9(29.0)		22(14.6)	2 (50.0)		21(22.1)	3(27.3)	
Yes	82(77.4)	35(79.5)	47(75.8)		60(80.0)	22(71.0)		60(85.4)	22(50.0)		74(77.9)	8(72.7)	
Lymphatic invasion				<0.0001			0.0065			0.8139			1.0000
Negative	38(35.8)	26(59.1)	12(19.4)		33(44.0)	5(16.1)		30(36.6)	8(33.3)		34(35.8)	4(36.4)	
Positive	68(64.2)	18(40.1)	50(80.6)		42(56.0)	26(83.9)		52(63.4)	16(66.7)		61(64.2)	7(63.6)	
UICC stage				0.0044			0.0010			0.1912			0.4757
IA/IB/IIA	28(26.4)	18(40.9)	10(16.1)		13(17.3)	15(48.4)		19(23.2)	9(37.5)		24(25.3)	4(36.4)	
IIB	78(73.6)	26(59.1)	52(83.9)		62(82.7)	16(51.6)		63(76.8)	15(62.5)		71(74.7)	7(63.6)	

* A P<0.05 was considered statistically significant.

**Table 2:** Associations of GLUD1, GOT1 expression with clinicopathological parameters.

Parameters	No. of cases (%)	GLUD1 in tumor			GLUD1 in stroma			GOT1 in tumor			GOT1 in stroma		
		Negative (n = 83) (%)	Positive (n = 23) (%)	P-value	Negative (n = 78) (%)	Positive (n = 28) (%)	P-value	Negative (n = 27) (%)	Positive (n = 79) (%)	P-value	Negative (n = 14) (%)	Positive (n = 92) (%)	P-value
Age				0.9432			1.0000			0.3744			0.4024
<61 years	50(47.2)	39(47.0)	11(47.8)		37(47.4)	13(46.4)		15(55.56)	35(44.3)		5(35.7)	45(48.9)	
≥61 years	56(52.8)	44(53.0)	12(52.2)		41(52.6)	15(53.6)		12(44.44)	44(55.7)		9(64.3)	47(51.1)	
Gender				0.7651			0.8253			0.8270			0.7814
Male	57(53.8)	44(53.0)	13(56.5)		43(55.1)	14(50.0)		14(81.5)	43(54.4)		7(50.0)	50(54.3)	
Female	49(46.2)	39(47.0)	10(43.5)		35(44.9)	14(50.0)		13(18.5)	36(45.6)		7(50.0)	42(45.7)	
Diabetes mellitus or hyperglycemia				0.8389			1.0000			0.4786			0.3792
No	35(33.0)	27(32.5)	8(34.8)		26(33.3)	9(32.1)		7(25.9)	28(35.4)		3(21.4)	32(34.8)	
Yes	71(67.0)	56(67.5)	15(65.2)		52(66.7)	19(67.9)		20(74.1)	51(64.6)		11(78.6)	60(65.2)	
Tumor Size				0.0086			0.4199			0.7814			0.1463
≤2.0cm	21(19.8)	12(14.5)	9(39.1)		14(17.9)	7(25.0)		6(22.22)	15(19.0)		5(35.7)	16(17.4)	
>2.0cm	85(80.2)	71(85.5)	14(60.9)		64(82.1)	21(75.0)		21(77.78)	64(81.0)		9(64.3)	76(82.6)	
Tumor Location				0.1486			0.7878			0.4049			0.7316
Head	85(80.2)	69(83.1)	16(69.6)		63(80.7)	22(78.6)		20(74.1)	65(82.3)		12(85.7)	73(79.3)	
Body/tail	21(19.8)	14(16.9)	7(30.4)		15(19.3)	6(21.4)		7(25.9)	14(17.7)		2(14.3)	19(20.7)	
Differentiation				0.8045			0.9473			0.0238			0.9773
Well	38(35.9)	31(37.3)	7(30.4)		28(35.9)	10(35.7)		7(25.9)	31(39.2)		5(35.7)	33(35.9)	
Moderate	40(37.7)	31(37.3)	9(39.1)		30(38.5)	10(35.7)		9(33.3)	31(39.2)		5(35.7)	35(38.0)	
Poor	28(26.4)	21(25.4)	7(30.5)		20(25.6)	8(28.6)		11(40.7)	17(21.5)		4(28.6)	24(26.1)	
Marginal status				0.5639			0.6038			0.1078			0.0743
R0	83(78.3)	66(79.5)	17(73.9)		62(79.5)	21(75.0)		18(66.7)	65(82.3)		8(57.1)	75(81.5)	
R1	23(21.7)	17(20.5)	6(26.1)		16(20.5)	7(25.0)		9(33.3)	14(17.7)		6(42.9)	17(18.5)	
Nerve Infiltration				1.0000			0.2796			1.0000			0.0087
Negative	22(20.8)	17(20.5)	5(21.7)		14(17.9)	8(28.6)		5(18.5)	17(21.5)		7(50.0)	15(16.3)	
Positive	84(79.2)	66(79.5)	18(78.3)		64(82.1)	20(71.4)		22(81.5)	62(78.5)		7(50.0)	77(83.7)	
Vascular invasion				0.5846			0.4331			0.0384			0.3005
No	24(22.6)	20(24.1)	4(17.4)		16(20.5)	8(28.6)		10(37.0)	14(17.7)		5(35.7)	19(20.7)	
Yes	82(77.4)	63(75.9)	19(82.6)		62(79.5)	20(71.4)		17(63.0)	65(82.3)		9(64.3)	73(79.3)	
Lymphatic invasion				0.6282			0.1780			0.0367			0.0812
Negative	38(35.8)	31(37.3)	7(30.4)		31(39.7)	7(25.0)		5(18.5)	33(41.8)		2(14.3)	36(39.1)	
Positive	68(64.2)	52(62.7)	16(69.6)		47(60.3)	21(75.0)		22(81.5)	46(58.2)		12(85.7)	56(60.9)	
UICC stage				0.6212			0.2312			0.3449			0.1342
IA/IB/IIA	28(26.4)	21(25.3)	7(30.4)		23(29.5)	5(17.9)		9(33.3)	19(24.1)		6(42.9)	22(23.9)	
IIB	78(73.6)	62(74.7)	16(69.6)		55(70.5)	23(82.1)		18(66.7)	60(75.9)		8(57.1)	70(76.1)	

**Table 3:** Associations of BNIP3, p62 expression with clinicopathological parameters.

Parameters	No. of cases (%)	BNIP3 in tumor			BNIP3 in stroma			Cytoplasmic p62 in tumor			Nuclear p62 in tumor			Nuclear p62 in stroma		
		Negative (n = 75) (%)	Positive (n = 31) (%)	P-value	Negative (n = 90) (%)	Positive (n = 16) (%)	P-value	Negative (n = 39) (%)	Positive (n = 67) (%)	P-value	Negative (n = 68) (%)	Positive (n = 38) (%)	P-value	Negative (n = 72) (%)	Positive (n = 34) (%)	P-value
Age				0.6696			1.0000			0.3191			1.0000			0.6829
<61 years	50(47.2)	34(45.3)	16(51.6)		42(46.7)	8(50.0)		21(53.8)	29(43.3)		32(47.1)	18(47.4)		35(48.6)	15(44.1)	
≥61 years	56(52.8)	41(54.7)	15(48.4)		48(53.3)	8(50.0)		18(46.2)	38(56.7)		36(52.9)	20(52.6)		37(51.4)	19(55.9)	
Gender				1.0000			0.5883			0.6920			0.2229			0.6779
Male	57(53.8)	40(53.3)	17(54.8)		47(52.2)	10(62.5)		22(56.4)	35(52.2)		40(58.8)	17(44.7)		40(55.6)	17(50.0)	
Female	49(46.2)	35(46.7)	14(45.2)		43(47.8)	6(37.5)		17(43.6)	32(47.8)		28(41.2)	21(55.3)		32(44.4)	17(50.0)	
Diabetes mellitus or hyperglycemia				0.4975			0.7746			0.3969			0.3892			0.3810
No	35(33.0)	23(30.7)	12(38.7)		29(32.2)	6(37.5)		15(38.5)	20(29.9)		20(29.4)	15(39.5)		26(36.1)	9(26.5)	
Yes	71(67.0)	52(69.3)	19(61.3)		61(67.8)	10(62.5)		24(61.5)	47(70.1)		48(70.6)	23(60.5)		46(63.9)	25(73.5)	
Tumor Size				0.7892			0.5172			0.6150			0.6115			0.0173
≤2.0cm	21(19.8)	14(18.7)	7(22.6)		17(18.9)	4(25.0)		9(23.1)	12(17.9)		13(19.1)	8(21.1)		19(13.9)	2(32.4)	
>2.0cm	85(80.2)	61(81.3)	24(77.4)		73(81.1)	12(75.0)		30(76.9)	55(82.1)		55(80.9)	30(78.9)		53(86.1)	32(67.6)	
Tumor Location				0.1791			0.0835			1.0000			0.8047			1.0000
Head	85(80.2)	63(84.0)	22(71.0)		75(83.3)	10(62.5)		31(79.5)	54(80.6)		55(80.9)	30(78.9)		58(80.6)	27(79.4)	
Body/tail	21(19.8)	12(16.0)	9(29.0)		15(16.7)	6(37.5)		8(20.5)	13(19.4)		13(19.1)	8(21.1)		14(19.4)	7(20.6)	
Differentiation				0.0155			0.2015			0.9109			0.2332			0.2560
Well	38(35.9)	31(41.3)	7(22.6)		33(36.7)	5(31.2)		15(38.5)	23(34.3)		28(41.2)	10(26.3)		29(40.3)	9(26.5)	
Moderate	40(37.7)	30(40.0)	10(32.2)		31(34.4)	9(56.3)		14(35.9)	26(38.8)		25(36.8)	15(39.5)		27(37.5)	13(38.2)	
Poor	28(26.4)	14(18.7)	14(45.2)		26(28.9)	2(12.5)		10(25.6)	18(26.9)		15(22.0)	13(34.2)		16(22.2)	12(35.3)	
Marginal status				0.6055			0.0010			0.0132			0.8069			1.0000
R0	83(78.3)	60(80.0)	23(74.2)		76(84.4)	7(45.8)		25(64.1)	58(86.6)		54(79.4)	29(76.3)		56(77.8)	27(79.4)	
R1	23(21.7)	15(20.0)	8(25.8)		14(15.6)	9(56.3)		14(35.9)	9(13.4)		14(20.6)	9(23.7)		16(22.2)	7(20.6)	
Nerve Infiltration				0.7954			0.1830			0.2137			0.1390			0.4418
Negative	22(20.8)	15(20.0)	7(22.6)		21(23.3)	1(6.3)		11(28.2)	11(16.4)		11(20.6)	11(28.9)		17(23.6)	5(14.7)	
Positive	84(79.2)	60(80.0)	24(77.4)		69(76.7)	15(93.7)		28(71.8)	56(83.6)		57(79.4)	27(71.1)		55(76.4)	29(85.3)	
Vascular invasion				0.1358			0.3518			0.6336			0.3331			0.3207
No	24(22.6)	14(18.7)	10(32.3)		19(21.1)	5(31.3)		10(25.6)	14(20.9)		13(19.1)	11(28.9)		14(22.2)	10(29.4)	
Yes	82(77.4)	61(81.3)	21(67.7)		71(78.9)	11(68.7)		29(74.4)	53(79.1)		55(80.9)	27(71.1)		58(77.8)	24(70.6)	
Lymphatic invasion				0.8241			0.7823			1.0000			1.0000			0.6685
Negative	38(35.8)	26(34.7)	12(38.7)		33(36.7)	5(31.3)		14(35.9)	24(35.8)		24(35.3)	14(36.8)		27(37.5)	11(32.3)	
Positive	68(64.2)	49(65.3)	19(61.3)		57(63.3)	11(68.7)		25(64.1)	43(64.2)		44(64.7)	24(63.2)		45(62.5)	23(67.6)	
UICC stage				0.0649			0.2751			0.1719			0.8186			0.4797
IA/IB/IIA	28(26.4)	16(21.3)	12(38.7)		22(24.4)	6(37.5)		7(17.9)	21(31.3)		19(27.9)	9(23.7)		21(29.2)	7(20.6)	
IIB	78(73.6)	59(78.7)	19(61.3)		68(75.6)	10(62.5)		32(82.1)	46(68.7)		49(72.1)	29(76.3)		51(70.8)	27(79.4)	

**Table 4:** Associations of LC3, Beclin-1 expression with clinicopathological parameters.

Parameters	No. of cases (%)	LC3 in tumor			LC3 in stroma			Cytoplasmic Beclin-1 in tumor			Nuclear Beclin-1 in tumor		
		Negative (n = 43) (%)	Positive (n = 63) (%)	P-value	Negative (n = 87) (%)	Positive (n = 19) (%)	P-value	Negative (n = 80) (%)	Positive (n = 26) (%)	P-value	Negative (n = 75) (%)	Positive (n = 31) (%)	P-value
Age				0.8440			0.8005			0.8226			1.0000
<61 years	50(47.2)	21(48.8)	29(46.0)		42(48.3)	8(42.1)		37(46.2)	13(50.0)		35(46.7)	15(48.4)	
≥61 years	56(52.8)	22(51.2)	34(54.0)		45(51.7)	11(57.9)		43(53.8)	13(50.0)		40(53.3)	16(51.6)	
Gender				0.1651			1.0000			0.4975			0.1370
Male	57(53.8)	27(62.8)	30(47.6)		47(54.0)	10(52.6)		45(56.2)	12(46.2)		44(58.7)	13(41.9)	
Female	49(46.2)	16(37.2)	33(52.4)		40 (46.0)	9(47.4)		35(43.8)	14(53.8)		31(41.3)	18(58.1)	
Diabetes mellitus or hyperglycemia				0.0205			0.2869			0.4837			1.0000
No	35(33.0)	20(46.5)	15(23.8)		31(35.6)	4(21.1)		28(35.0)	7(26.9)		25(33.3)	10(32.3)	
Yes	71(67.0)	23(53.5)	48(76.2)		56(64.4)	15(78.9)		52(65.0)	19(73.1)		50(66.7)	21(67.7)	
Tumor Size				0.0453			0.1125			1.0000			0.7892
≤2.0cm	21(19.8)	13(30.2)	8(12.7)		20(23.0)	1(5.3)		16(20.0)	5(19.2)		14(18.7)	7(22.6)	
>2.0cm	85(80.2)	30(69.8)	55(87.3)		67(77.0)	18(94.7)		64(80.0)	21(80.8)		61(81.3)	24(77.4)	
Tumor Location				0.3209			0.7592			1.0000			0.6041
Head	85(80.2)	37(86.0)	48(76.2)		69(85.1)	16(57.9)		64(80.0)	21(80.8)		59(78.7)	26(83.9)	
Body/tail	21(19.8)	6(14.0)	15(23.8)		18(14.9)	3(42.1)		16(20.0)	5(19.2)		16(21.3)	5(16.1)	
Differentiation				0.0040			< 0.0001			0.5450			0.3704
Well	38(35.9)	23(53.5)	15(23.8)		34(39.1)	4(21.1)		30(37.5)	8(30.8)		30(40.0)	8(25.8)	
Moderate	40(37.7)	14(32.5)	26(41.3)		33(37.9)	7(36.8)		31(38.8)	9(34.6)		26(34.7)	14(45.2)	
Poor	28(26.4)	6(14.0)	22(34.9)		20(23.0)	8(42.1)		19(23.7)	9(34.6)		19(25.3)	9(29.0)	
Marginal status				0.6338			0.0010			0.2722			0.0379
R0	83(78.3)	35(81.4)	48(76.2)		74(85.1)	9(47.4)		65(81.3)	18(69.2)		63(84.0)	20(64.5)	
R1	23(21.7)	8(18.6)	15(23.8)		13(14.9)	10(52.6)		15(18.8)	8(30.8)		12(16.0)	11(35.5)	
Nerve Infiltration				0.1501			0.2187			0.5813			0.7954
Negative	22(20.8)	12(27.9)	10(15.8)		16(18.4)	6(31.6)		18(22.5)	4(15.4)		15(20.0)	7(22.6)	
Positive	84(79.2)	31(72.1)	53(84.1)		71(81.6)	13(68.4)		62(77.5)	22(84.6)		60(80.0)	24(77.4)	
Vascular invasion				0.0044			0.0015			0.7896			0.4445
No	24(22.6)	16(37.2)	8(12.7)		14(16.1)	10(52.6)		19(23.8)	5(19.2)		19(25.3)	5(16.1)	
Yes	82(77.4)	27(62.8)	55(87.3)		73(83.9)	9(47.4)		61(76.2)	21(80.8)		56(74.7)	26(83.9)	
Lymphatic invasion				0.0385			0.0633			0.0175			0.1883
Negative	38(35.8)	10(23.3)	28(44.4)		35(40.2)	3(15.8)		34(42.5)	4(15.4)		30(40.0)	8(25.8)	
Positive	68(64.2)	33(76.7)	35(55.6)		52(59.8)	16(84.2)		46(57.5)	22(84.6)		45(60.0)	23(74.2)	
UICC stage				1.0000			1.0000			0.3103			0.4684
IA/IB/IIA	28(26.4)	11(25.6)	17(27.0)		23(26.4)	5(26.3)		19(23.8)	9(34.6)		18(24.0)	10(32.3)	
IIB	78(73.6)	32(74.4)	46(73.0)		64(73.6)	14(73.7)		61(76.2)	17(65.4)		57(76.0)	21(67.7)	

### Correlation between tumor metabolic phenotypes and clinicopathological parameters, mitochondrial function and autophagic status

**Fig 1:**
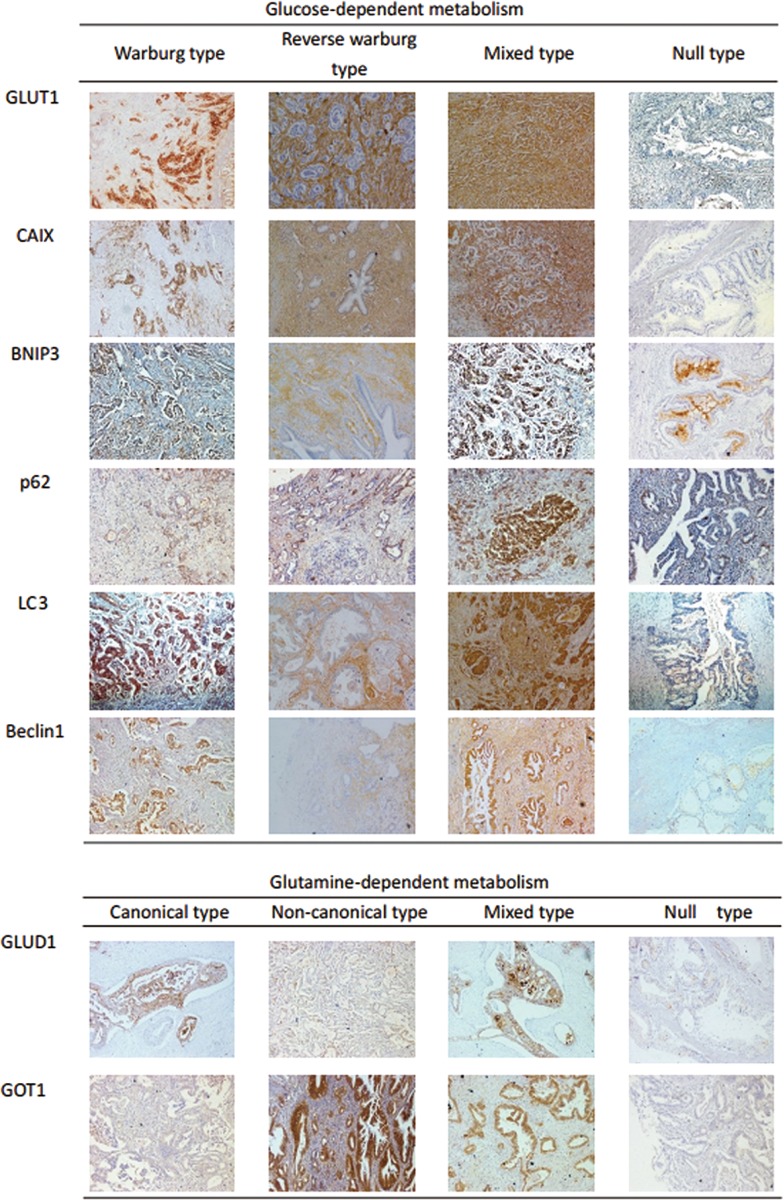
Typical immunohistochemical labeling of each metabolism-related proteins according to metabolic phenotypes in pancreatic cancer.

Classifications of tumor metabolic phenotypes were summarized according to previous reports (Table 2 in S2 File) and representative immunohistochemical labeling profiles were shown in Fig. 1 [13,14]. Warburg type refers to a phenotype in which only tumor cells show increased glycolysis and glycolysis takes place in stroma not tumor in reverse Warburg type. Both tumor cells and stromal cells are glycolytic in mixed type and nonglycolytic in null type. The correlations between the metabolic phenotypes of pancreatic cancer and clinicopathological parameters were summarized in Table 5. The total 106 tumor samples were divided into following four types according to their glycolysis-related proteins expression: Warburg type (n = 46, 43.4%), reverse Warburg type (n = 15, 14.2%), mixed type (n = 16, 15.1%), null type (n = 29, 27.3%). Warburg type of glucose-dependent metabolism had a highest percentage of tumors with nerve infiltration (P = 0.0003), UICC stage (P = 0.0004). Mixed type of glucose-dependent metabolism comprised the highest percentage of tumors located in the body/tail (P = 0.0041), with positive marginal status (P<0.0001), and with lymphatic invasion (P<0.0001). Reverse Warburg type had a highest percentage of tumors located in head (P = 0.0041), with absence of nerve infiltration (P = 0.0003). Null type of glucose-dependent metabolism comprised highest percentage of tumors with negative marginal status (P<0.0001) and absence of lymphatic invasion (P<0.0001).

**Table 5:** Clinicopathological characteristics of patients according to metabolic phenotype.

Parameters	No. of cases (%)	Glucose-dependent metabolism					Glutamine-dependent metabolism					
		Warburg type (n = 46) (%)	Reverse Warburgtype (n = 15 (%)	Mixed type (n = 16) (%)	Null type (n = 29) (%)	P value	Canonical type (n = 18) (%)		Non-canonical type (n = 74) (%)	Mixed type(n = 5) (%)	Null type (n = 9) (%)	P-value
Age						0.8886						0.2230
<61 years	50(47.2)	22(47.8)	6(40.0)	7(43.8)	15(51.7)		8(44.4)		32(43.2)	3(60.0)	7(77.8)	
≥61 years	56(52.8)	24(52.2)	9(60.0)	9(56.2)	14(48.3)		10(55.6)		42(56.8)	2(40.0)	2(22.2)	
Gender						0.3488						0.0544
Male	57(53.8)	25(54.3)	5(33.3)	10(62.5)	17(58.6)		8(44.4)		38(51.3)	5(100.0)	6(66.7)	
Female	49(46.2)	21(45.7)	10(66.7)	6(37.5)	12(41.4)		10(55.6)		36(48.7)	0(0)	3(33.3)	
Diabetes mellitus or hyperglycemia						0.1948						0.5164
No	35(33.0)	14(30.4)	4(26.7)	9(56.3)	8(27.6)		5(27.8)		25(33.8)	3(60.0)	2(22.2)	
Yes	71(67.0)	32(69.6)	11(73.3)	7(43.7)	21(72.4)		13(72.2)		49(66.2)	2(40.0)	7(77.8)	
Tumor Size						0.1153						0.0163
≤2.0cm	21(19.8)	8(17.4)	6(40.0)	4(25.0)	3(10.3)		6(33.3)		12(16.2)	3(60.0)	0(0)	
>2.0cm	85(80.2)	38(82.6)	9(60.0)	12(75.0)	26(89.7)		12(66.7)		62(83.8)	2(40.0)	9(100.0)	
Tumor Location						0.0041						0.4739
Head	85(80.2)	42(91.3)	14(93.3)	9(56.3)	20(69.0)		12(66.7)		61(82.4)	4(80.0)	8(88.9)	
Body/tail	21(19.8)	4(8.7)	1(6.7)	7(43.7)	9(31.0)		6(33.3)		13(17.6)	1(20.0)	1(11.1)	
Differentiation						0.2009						0.4849
Well	38(35.9)	19(41.3)	3(20.0)	4(25.5)	12(41.4)		5(27.8)		29(39.2)	2(40.0)	2(22.2)	
Moderate	40(37.7)	19(41.3)	5(33.3)	5(31.3)	11(37.9)		7(38.9)		29(39.2)	2(40.0)	2(22.2)	
Poor	28(26.4)	8(17.4)	7(46.7)	7(43.2)	6(20.7)		6(33.3)		16(21.6)	1(20.0)	5(55.6)	
Marginal status						<0.0001						<0.0001
R0	83(78.3)	36(78.3)	13(86.7)	6(37.5)	28(96.6)			17(94.4)	65(87.8)	0(0)	1(11.1)	
R1	23(21.7)	10(21.7)	2(13.3)	10(62.5)	1(3.4)			1(5.6)	9(12.2)	5(100.0)	8(88.9)	
Nerve Infiltration						0.0003						0.7601
Negative	22(20.8)	4(8.7)	9(60.0)	4(25.0)	5(17.2)			3(16.7)	15(20.3)	2(40.0)	2(22.2)	
Positive	84(79.2)	42(91.3)	6(40.0)	12(75.0)	24(82.8)			15(83.3)	59(79.7)	3(60.0)	7(77.8)	
Vascular invasion						0.4958						0.0073
Negative	24(22.6)	9(19.6)	3(20.0)	6(37.5)	6(20.7)			4(22.2)	14(18.9)	0(0)	6(66.7)	
Positive	82(77.4)	37(80.4)	12(80.0)	10(62.5)	23(79.3)			14(77.8)	60(81.1)	5(100.0)	3(33.3)	
Lymphatic invasion						<0.0001						0.0802
Negative	38(35.8)	10(21.7)	3(20.0)	2(12.5)	23(79.3)			5(27.8)	31(41.9)	2(40.0)	0(0)	
Positive	68(64.2)	36(78.3)	12(80.0)	14(87.5)	6(20.7)			13(72.2)	43(58.1)	3(60.0)	9(100.0)	
UICC stage						0.0004						0.4847
IA/IB/IIA	28(26.4)	3(6.5)	8(53.3)	7(43.8)	10(34.5)			5(27.8)	17(23.0)	2(40.0)	4(44.4)	
IIB	78(73.6)	43(93.5)	7(46.7)	9(56.2)	19(65.5)			13(72.2)	57(77.0)	3(60.0)	5(55.6)	

Mitochondrial function and autophagic status were further assessed according to subtype of glucose-dependent metabolism. We found that Warburg type comprised a highest percentage of tumors with activated autophagy (P = 0.0167), mixed type and reverse Warburg type with activated autophagic status in stroma (P = 0.0002), reverse Warburg type with nonacitivated autophagic status in tumor (P = 0.0167), and null type with nonactivated autophagic status in stroma (P = 0.0002) (Table 6).

The total 106 tumor samples were also divided into four subtypes of glutamine-dependent metabolism, including canonical type (n = 18, 17.0%), non-canonical type (n = 74, 69.8%), mixed type (n = 5, 4.7%) and null type (n = 9, 8.5%) (Table 5). The canonical type refers to a phenotype in which tumors rely on glutamate dehydrogenase (GLUD1) for glutamine utilization; whereas non-canonical type refers to a phenotype in which tumor rely on aspartate transaminase (GOT1) for glutamine utilization [9,15]. The mixed type of glutamine-dependent metabolism refers to a phenotype in which tumors metabolize glutamine through GLUD1 and GOT1, and null type refers to a phenotype in which tumors metabolize glutamine by neither GLUD1 nor GOT1. Canonical type had a highest percentage of tumors with negative marginal status (P<0.0001), whereas mixed type of glutamine-dependent metabolism comprised the highest percentage of tumors with positive marginal status (P<0.0001), vascular invasion (p = 0.0073). A significant association was found between the null type of glutamine-dependent metabolism and tumors with larger size (>2.0cm) (p = 0.0163). Moreover, tumors of non-canonical type and mixed type had a significantly activated autophagic status in tumor (P = 0.0034), and non-canonical type comprised the highest percentage of stroma with functional mitochondrial status (P = 0.0046) (Table 6).

**Table 6:** Mitochondrial function and autophagic status according to metabolic phenotype.

Parameters	No. of cases (%)	Glucose-dependent metabolism					Glutamine-dependent metabolism					
		Warburg type (n = 46) (%)	Reverse Warburg type (n = 15 (%)	Mixed type (n = 16) (%)	Null type (n = 29) (%)	P value	Canonical type (n = 18) (%)		Non-canonical type (n = 74) (%)	Mixed type(n = 5) (%)	Null type (n = 9) (%)	P-value
Tumor mitochondrial status						0.2642						0.0999
Dysfunctional	31(29.2)	12(26.1)	7(46.7)	6(37.5)	6(20.7)			8(44.4)	17(23.0)	1(20.0)	5(55.6)	
Functional	75(70.8)	34(73.9)	8(53.3)	10(62.5)	23(79.3)			10(55.6)	57(77.0)	4(80.0)	4(44.4)	
Stroma mitochondrial status						0.1356						0.0046
Dysfunctional	16(15.1)	4(8.7)	5(33.3)	2(12.5)	5(17.2)			3(16.7)	6(8.1)	3(60.0)	4(44.4)	
Functional	90(84.9)	42(91.3)	10(66.7)	14(87.5)	24(82.8)			15(83.3)	68(91.9)	2(40.0)	5(55.6)	
Tumor autophagic status						0.0167						0.0034
Activated	57(53.8)	30(65.2)	3(20.0)	7(43.7)	17(58.6)			5(27.8)	44(59.5)	5(100.0)	3(33.3)	
Nonactivated	49(46.2)	16(34.8)	12(80.0)	9(56.3)	12(41.4)			13(72.2)	30(40.0)	0(0)	6(66.7)	
Stroma autophagic status						0.0002						0.1629
Activated	18(17.0)	5(10.9)	6(37.5)	7(43.7)	0(0)			4(11.1)	13(17.6)	1(20.0)	0(0)	
Nonactivated	88(83.0)	41(89.1)	10(62.5)	9(56.3)	29(100.0)			14(88.9)	61(82.4)	4(80.0)	9(100.0)	

### Impact of clinicopathological parameters and metabolism-related proteins on patient prognosis

Univariate analysis was performed to explore the relation of the clinicopathological parameters/metabolism-related proteins and overall survivals (Table 7 and Figure B in S1 File). Higher histological grade (P = 0.041) was significantly associated with shorter overall survival. Longer overall survival was associated with high expression of BNIP3 in tumor (p = 0.010). Shorter overall survival was associated with high expression of GLUT1 in tumor (P = 0.002), GOT1 in tumor (p = 0.030), and LC3 in tumor (p = 0.009). There were borderline significant differences in nerve infiltration with regard to overall survival (P = 0.075).

**Table 7:** Clinicopathological Parameters/the expression of metabolism-related proteins and overall survival by log-rank test.

Variables	Number of patients/death	Overall survival		Variables	Number of patients/death	Overall survival	
		Mean survival(95% CI), months	P-value			Mean survival(95% CI), months	P-value
Age			0.272	Marginal status			0.314
<61 years	50/35	17(13 to 21)		R0	83/63	20(16 to 25)	
≥61 years	56/44	22(17 to 28)		R1	23/16	17(10 to 24)	
Gender			0.268	Nerve Infiltration			0.075
Male	57/45	18(13 to 22)		Negative	22/18	14(10 to 17)	
Female	49/34	23(17 to 29)		Positive	84/61	22(17 to 26)	
Diabetes mellitus or hyperglycemia			0.840	Vascular invasion			0.523
No	35/25	20(13 to 27)		No	24/19	18(16 to 27)	
Yes	71/54	20(16 to 25)		Yes	82/60	24(17 to 29)	
Tumor Size			0.562	Lymphatic invasion			0.373
≤2.0cm	21/17	23(14 to 31)		Negative	38/30	18(12 to 24)	
>2.0cm	85/62	19(15 to 23)		Positive	68/49	21(16 to 25)	
Tumor Location			0.243	UICC Stage			0.012
Head	85(80.2)	21(15 to 28)		IA/IB/IIA	28/17	20(15 to 27)	
Body/tail	21(19.8)	25(19 to 33)		IIB	78/62	13(8 to 15)	
Differentiation			0.041	GLUT1 in tumor			0.002
Well	38/28	21(18 to27)		Negative	44/29	28(22 to 34)	
Moderate	40/27	16(10 to 22)		Positive	62/50	17(13 to 21)	
Poor	28/24	14(9 to 19)					
GLUT1 in stroma			0.520	Cytoplasmic p62 in tumor			0.310
Negative	75/56	23(18 to 28)		Negative	39/29	23(17 to 30)	
Positive	31/23	19(14 to 24)		Positive	67/50	21(17 to 26)	
CAIX in tumor			0.807	Cytoplasmic p62 in tumor			0.698
Negative	82/61	22(15 to 30)		Negative	39/29	23(17 to 30)	
Positive	24/18	23(15 to 30)		Positive	67/50	21(17 to 26)	
CAIX in stroma			0.234	Nuclear p62 in stroma			0.983
Negative	95/69	22(18 to 26)		Negative	72/53	21(16 to 28)	
Positive	11/10	17(8 to 26)		Positive	34/26	22(17 to 27)	
GLUD1 in tumor			0.783	LC3 in tumor			0.009
Negative	83/60	25(18 to 33)		Negative	43/28	28(17 to 35)	
Positive	23/19	21(17 to 26)		Positive	63/51	17(13 to 20)	
GLUD1 in stroma			0.990	LC3 in stroma			0.691
Negative	78/57	22(18 to 27)		Negative	87/66	21(17 to 25)	
Positive	28/22	21(14 to 28)		Positive	19/13	25(14 to 36)	
GOT1 in tumor			0.030	BNIP3 in tumor			0.010
Negative	79/60	33(25 to 40)		Negative	75/58	21(16 to 25)	
Positive	27/19	18(14 to 22)		Positive	31/21	24(17 to 32)	
GOT1 in stroma			0.449	BNIP3 in stroma			0.991
Negative	14/8	29(15 to 42)		Negative	75/58	22(18 to 26)	
Positive	92/71	21(17 to 25)		Positive	31/21	23(15 to 31)	

### Impact of metabolism phenotypes on patient prognosis

**Fig 2:**
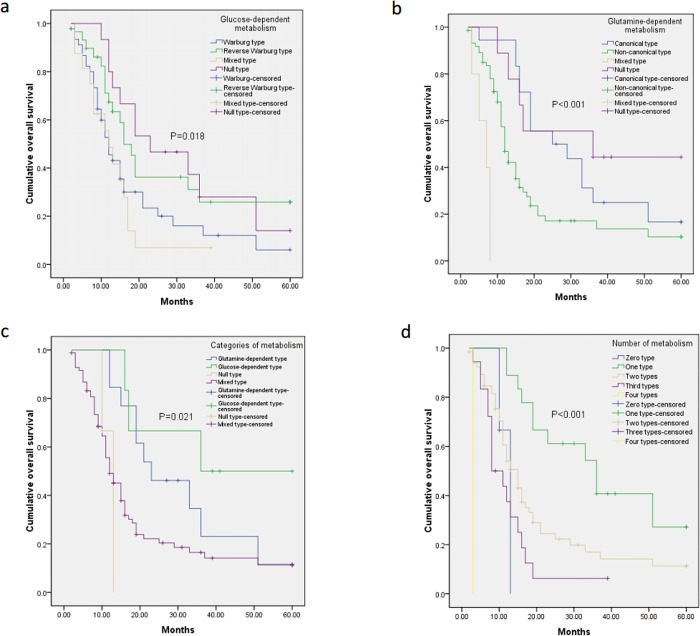
Overall survival curves according to the metabolic phenotypes of pancreatic cancer, including glucose-dependent metabolism (a), glutamine-dependent metabolism (b), combination of the two metabolism categories including glucose- and glutamine-dependent metabolism (c), and numbers of metabolism subtypes (d). We assumed that mixed types composed of two main types within glucose- and glutamine-dependent metabolism (i.e. Warburg effect and Reverse Warburg effect, Canonical type and Non-canonical type), and the null types were not composed of any of the two main types in glucose- and glutamine-dependent metabolism (i.e. neither Warburg effect nor Reverse Warburg effect, neither Canonical type nor Non-canonical type.

**Fig 3:**
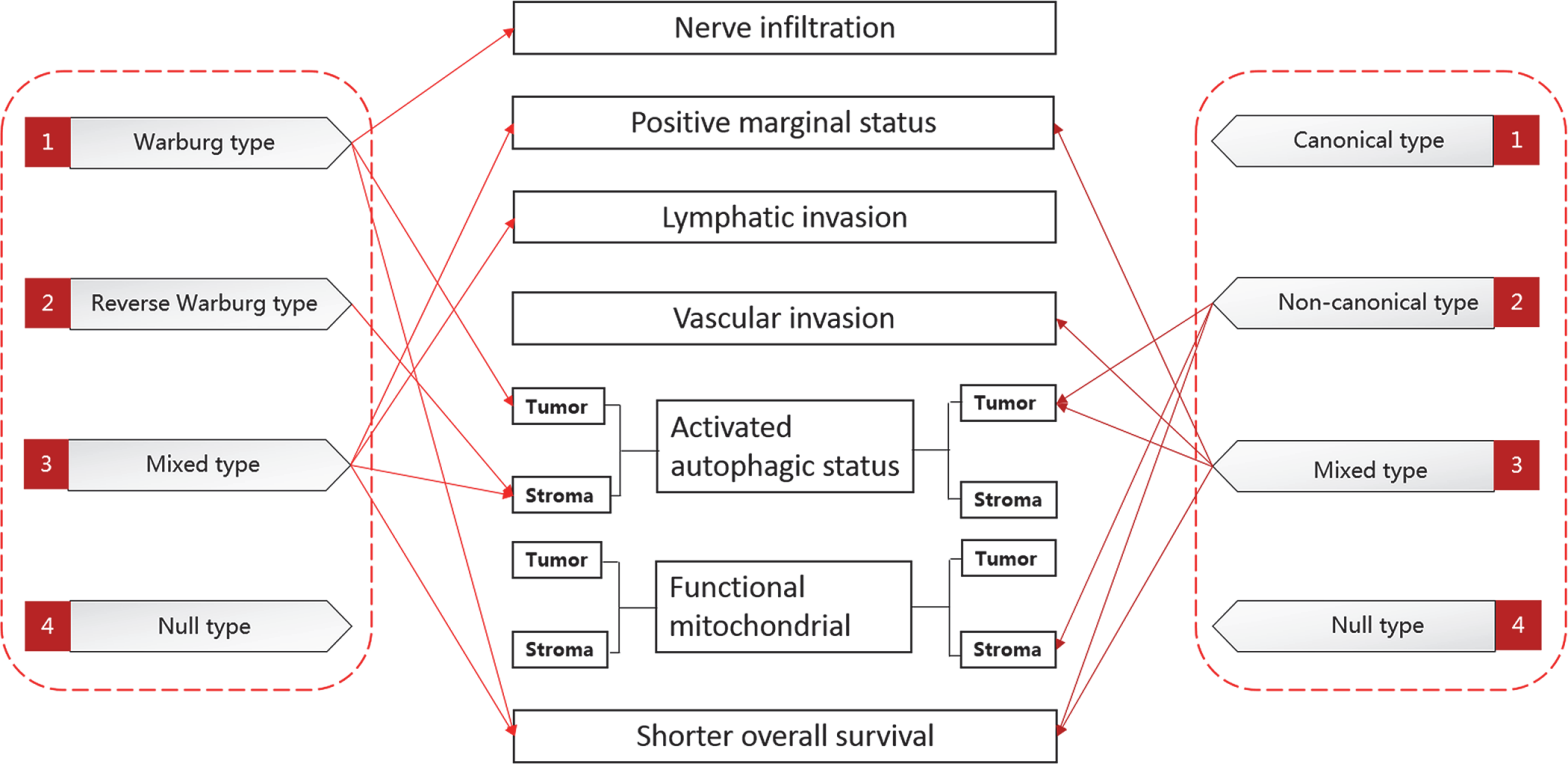
Schematic representation summing up the associations of various metabolic categories and subtypes with the specific associated parameters. A solid line with arrow means metabolic categories or subtypes have the highest percentage of the specific associated parameters.

The results of univariate analysis using log-rank tests of the metabolism phenotypes in relation to overall survival are presented in Table 8. Glucose-dependent metabolism status was significantly associated with overall survival (P = 0.018) (Fig. 2A). Then we performed pairwise comparison among the glucose-dependent metabolism subtypes, and found that Warburg type and mixed type of glucose-dependent metabolism were significantly associated with shorter overall survival (Warburg type vs. reverse Warburg type, P = 0.0340; Warburg type vs. Null type, P = 0.0480; Mixed type vs. reverse Warburg type, P = 0.0060; Mixed type vs. Null type, P = 0.0350). There was no difference between other pairs (Warburg type vs. Mixed type, P = 0.4880; Reverse Warburg type vs. null type, P = 0.648) (Table 3 in S2 File). We also found that glutamine-dependent metabolism status was significantly associated with overall survival (P<0.001) (Fig. 2B and Table 8). After pairwise test was performed, we found that canonical type was significantly associated with longer overall survival (Canonical type vs. Non-canonical type, p = 0.009; Canonical type vs. Mixed type, P<0.001). Non-canonical type was significantly associated with longer overall survival than mixed type (P<0.001), but shorter than null type (P = 0.028) and the canonical type (P = 0.0009). The mixed type of glutamine-dependent metabolism was significantly associated with shorter overall survival than null type (P<0.001). There was no difference between canonical type and null type (P = 0.453) (Table 4 in S2 File) (Fig. 3).

**Table 8:** Univariate analysis of metabolism phenotypes in pancreatic cancers and overall survival by log-rank test.

Metabolism phenotypes	Number of patients/death	Overall survival	
		Mean survival(95% CI), months	P-value
Glucose-dependent metabolic status			0.018
Warburg type	46/36	18(13 to 23)	
Reverse Warburg type	15/11	30(21 to 40)	
Mixed type	16/14	13(9 to 18)	
Null type	29/18	27(19 to 36)	
Glutamine-dependent metabolic status			<0.001
Canonical type	18/14	31(23 to 39)	
Non-canonical type	74/55	19(14 to 23)	
Mixed type	5/5	6(4 to 8)	
Null type	9/5	37(23 to 51)	
Categories of metabolism*			0.021
Glutamine-dependent metabolism	26/17	29(21 to 41)	
Glucose-dependent metabolism	6/4	37(22 to 45)	
Mixed type	71/57	19(15 to 23)	
Null type	3/1	51(39 to 65)	
No. of metabolism types^#^			<0.001
Zero type	3/1/	51(39 to 65)	
One type	31/20	29(17 to 35)	
Two types	53/41	21(16 to 26)	
Three types	18/16	12(8 to 16)	
Four types	1/1	3(3 to 3)	

* Glutamine-dependent type (tumors only rely on glutamine-dependent metabolism), glucose-dependent type (tumors only rely on glucose-dependent metabolism), mixed type (tumors rely on both glucose- and glutamine-dependent metabolism), and null type (tumors show no sign of glucose- or glutamine-dependent metabolism)

^#^Metabolism types refer to cross combination among glucose- and glutamine-dependent metabolisms. Mixed types were composed of two main types of metabolism (i.e. Warburg effect and Reverse Warburg effect, Canonical type and Non-canonical type), and the null types were not composed of zero type in glucose- and glutamine-dependent metabolism (i.e. neither Warburg effect nor Rev erse Warburg effect, neither Canonical type nor Non-canonical type).

In order to compare the two categories of metabolism in parallel, we classified the tumors into following groups according to the two categories of metabolism: glucose-dependent type (tumors only rely on glucose-dependent metabolism and have no sign of glutamine-dependent metabolism), glutamine-dependent type (tumors only rely on glutamine-dependent metabolism and show no sign of glucose-dependent metabolism), mixed type (tumors rely on both glucose- and glutamine-dependent metabolism), and null type (tumors show no sign of glucose- or glutamine-dependent metabolism) (Table 8). Most (n = 71, 67.0%) of the included tumors in present study were mixed type, followed by glutamine-dependent metabolism (n = 26, 24.5%), glucose-dependent metabolism (n = 6, 5.7%), and null type (n = 3, 2.8%). It suggested that most tumors utilized both glucose- and glutamine-dependent metabolism to survive under shortage of nutrients and oxygen. Then we conducted Kaplan-Meier survival analysis and found that categories of metabolism were significantly associated with overall survival of patients (P = 0.021) (Fig. 2C). However, after pairwise test was done, only the null type significantly linked with longer overall survival than glutamine- and glucose-dependent metabolism. Notably, there was no significant difference in overall survival between glucose-dependent metabolism and glutamine-dependent metabolism (P = 0.279). However, careful consideration should be given to these results due to lack of data in null type and glucose-dependent type (Table 5 in S2 File). We hypothesized that the tumor with mixed type or Warburg type of glucose-dependent metabolism are highly glycolytic, and tumors with non-canonical type and mixed type of glutamine-dependent metabolism are highly glutaminolytic. We found that highly glycolytic tumors were also more dependent on glutamine-dependent metabolism and vice versa (P<0.0001).

Next, we hypothesized that mixed types were composed of two main subtypes in glucose- and glutamine-dependent metabolism (i.e. Warburg type and Reverse Warburg type, Canonical type and Non-canonical type), and the null types were not composed of any of the two main types in glucose- and glutamine-dependent metabolism (i.e. neither Warburg type nor Reverse Warburg type, neither Canonical type nor Non-canonical type). Therefore, based on the types of glucose-dependent and glutamine-dependent metabolism, we classified the patients into 4 groups: zero type (n = 3, 2.8%), one type (n = 31, 29.2%), two types (n = 53, 50.0%), three types (n = 18, 17.0%), four types (n = 1, 0.9%) (Table 8). As indicated in Kaplan-Meier survival analysis, the overall survival of one-type group was significantly longer than two-type group (P = 0.039) and three-type group (P = 0.005) (Fig. 2D). Moreover, two-type group show a relatively longer overall survival than three-type group (P = 0.024). Statistical analysis comparing zero type or four type and other types was also performed, but the results needed to be taken careful consideration due to small number of patients. To sum up, the increasing numbers of the metabolism types robustly reflected major differences in survival outcome (Table 8 and Table 6 in S2 File).

### Multivariate analysis of relation between prognostic factors and patient survival

Finally, multivariate analysis was also performed when P value in univariate analysis was less than 0.1. Prognostic factors including tumor differentiation, nerve infiltration, expression of GLUT1 in tumor, GOT1 in tumor, expression of LC3 in tumor, expression of BNIP3 in tumor, glucose-dependent metabolism, glutamine-dependent metabolism, categories of metabolism and numbers of metabolism types in tumor were analyzed in Multivariate models using Cox proportional hazards analysis. The results showed that independent factors for overall survival were higher histological grade (hazard ration 1.734, P = 0.015), GLUT1 in tumor (hazard ration 1.781, P = 0.003), GOT1 in tumor (hazard ration 1.982, P = 0.001), LC3 in tumor (hazard ration 3.321, P = 0.001), glutamine-dependent metabolism (hazard ration 1.471, P = 0.041), glucose-dependent metabolism (hazard ration 1.689, P = 0.022), numbers of metabolism type (hazard ratio 1.806, P- = 0.031). However, expression of BNIP3 in tumor was significantly associated with better prognosis (hazard ration 0.049, P = 0.008) (Table 9).

**Table 9:** Multivariate Analysis of Prognostic Factors for Overall Survival.

Variable	Hazard Ratio	95% Confidence Limit		P-value
		Lower limit	Upper limit	
Tumor differentiation	1.734	1.231	2.563	0.015
Nerve infiltration	0.823	0.554	1.678	0.062
GLUT1 in tumor	1.781	1.024	2.843	0.003
GOT1 in tumor	1.982	1.237	2.561	0.001
LC3 in tumor	3.321	1.660	6.642	0.001
BNIP3 in tumor	0.409	0.233	0.718	0.008
Glutamine-dependent metabolism	1.417	1.014	1.979	0.041
Glucose-dependent metabolism	1.689	1.005	2.641	0.022
Categories of metabolism	1.245	0.746	1.892	0.384
No. of metabolism types	1.806	1.055	3.090	0.031

### Transcript level of metabolism-related genes in tumor according to tumor phenotype

In order to further confirm results from IHC above, QRT-PCR was performed to investigate expression of metabolism-related genes (Table 7 in S2 File) in tumor according to the tumor phenotype. The expression of SLC2A1(gene of GLUT1), BNIP3, BECN1(gene of Beclin1) and MAP1LC3A(gene of LC3) was higher in Warburg types, whereas the expression of GLUT1, CAIX, BNIP3, BECN1, MAP1LC3A and SQSTM1(gene of p62) were higher in mixed type of glucose-dependent metabolism than in controls. The expression of GLUD1 was higher in canonical type than controls, and expression of GOT1 was higher in non-canonical type and mixed type (Figure C in S1 File).

## Discussion

The prognosis of pancreatic cancer patients is still dismal, and the disease is obviously resistant to current therapy[16]. Therefore, there is a growing need for new strategies to enhance the antitumor effect of gemcitabine in pancreatic cancer, which is the standard chemotherapeutic agent in pancreatic cancer. Recently, there has been growing interest in understanding cancer altered metabolism and targeting cancer metabolism holds a promising strategy for cancer treatment[4,9,17–19]. Before such effective metabolic drugs come into use, metabolism phenotypes in individual patient with pancreatic cancer need to be clearly identified due to obvious heterogeneity in pancreatic cancer [20,21]. However, little is known about metabolic features in pancreatic cancer.

Therefore, the present study was conducted to profile the glucose-dependent and glutamine-dependent metabolism in patients with pancreatic cancer. To our best knowledge, our study was the first study that focused on profiling metabolism phenotypes in pancreatic cancer. In order to identify the metabolism phenotype of individual tumor specimen, we firstly investigated the differential expression of metabolism-related protein markers not only in tumor cells but also in stromal cells. Besides, we also investigate the autophagic status through p62, LC3, Beclin1 expression and mitochondrial function by BNIP3 expression. It was reported that p62 is shuttled between the nucleus and the cytosol continuously. p62 is required to recruit the large phosphoinositide-binding protein ALFY (autophagy-linked FYVE protein, also known as WDFY3)，which is predominantly located at nuclear, to cytoplasmic p62 bodies. ALFY, as well as p62, is required for formation and autophagic degradation of cytoplasmic ubiquitin-positive inclusions [22,23]. BNIP3 is a pro-apoptotic member of the Bcl-2 family induced under hypoxia and was employed to assess the mitochondrial function. It was recently shown that BNIP3 mediates mitochondrial dysfunction through activation of Bax or Bak, and induced mitochondrial permeability transition via its carboxy terminal tail. Therefore, overexpression of BNIP3 indicates the dysfunctional mitochondria [24,25]. Of note, stromal cells metabolize through glycolysis due to dysfunctional mitochondria and enhanced autophagy, while tumor cells metabolize through oxidative phosphorylation according to the theory of reverse Warburg effect [7,26,27]. As there is no data reporting the reverse Warburg effect in pancreatic cancer, the present study was carried out to verify the existence of reverse Warburg effect in pancreatic cancer in the meantime. We found that increased tumor expression of GLUT1 was associated with nerve infiltration, lymphatic invasion, UICC stage, whereas stoma expression of GLUT1 was associated with tumor dedifferentiation, positive marginal status, nerve infiltration, lymphatic invasion and UICC stage. Tumor expression of GLUD1 was associated with larger tumor size, while tumor expression of GOT1 was significantly linked with tumor dedifferentiation, vascular invasion, and lymphatic invasion. The results above proved the existence of reverse Warburg type in pancreatic cancer (defined when tumor is oxidative phosphorylation type and stroma is glycolytic with enhanced autophagy and dysfunctional mitochondrial status). According to the definition of Warburg and other types of glucose-dependent metabolism in breast cancer elsewhere [13,14], glucose-dependent metabolism phenotypes in pancreatic cancer were Warburg type>null type> mixed type > reverse Warburg type in order of frequency. Further, we found that each glucose-dependent metabolism subtypes has its own characteristics. Warburg type of glucose-dependent metabolism had a highest percentage of tumors with nerve infiltration, UICC stage, and activated autophagic status in tumor. Mixed type of glucose-dependent metabolism comprised the highest percentage of tumors located in the body/tail, with positive marginal status, with lymphatic invasion, with activated autophagic status in stroma. Reverse Warburg type had a highest percentage of tumors located in head, with absence of nerve infiltration, and with nonacitivated autophagic status in tumor. Null type of glucose-dependent metabolism comprised highest percentage of tumors with negative marginal status, absence of lymphatic invasion, nonactivated autophagic status in stroma. Moreover, Warburg type and mixed type had a significantly poorer prognosis than reverse Warburg and null type. In summary, the Warburg type and mixed type comprised of more metabolically active, biologically aggressive and poorer prognostic tumors, whereas the reverse Warburg type and null type consisted of metabolically inactive, biologically nonaggressive and better prognostic tumors.

Besides glucose-dependent metabolism, we also assessed the glutamine-dependent metabolism, which has been proved to be critical for pancreatic cancer cell growth. As the most abundant amino acid in cytoplasm, glutamine plays an important part in marcromolecular synthesis by fueling TCA cycle and ATP production, nitrogen for nucleotide, nonessential amino acid, and hexosamine biosynthesis [18,28]. Recent evidence showed that pancreatic cancer cells mostly utilizes glutamic-oxaloacetic transaminase (including GOT1) (Non-canonical type) other than glutamate dehydrogenase (GLUD1) (Canonical type) for converting into alpha-ketoglutarate to fuel TCA cycle, which is obviously different from other kinds of tumors and normal cells. Son and his colleagues had proved that GOT1 mediates the utilization of glutamine in pancreatic cancer; oncogenic KRAS coordinates the shift from canonical type to non-canonical type of glutamine-dependent metabolism by increasing expression of GOT1 and decreasing expression of GLUD1 [9,15,29,30]. To our best knowledge, the profiling of glutamine-dependent metabolism still lacked in pancreatic cancer. Therefore, we evaluated this kind of metabolism in human samples. The present study consisted of highest percentage of non-canonical type, followed by canonical type, mixed type and null type. Mixed type of glutamine-dependent metabolism comprised the highest percentage of tumors with positive marginal status, vascular invasion. Tumors of non-canonical type and mixed type had a significantly activated autophagic status in tumor, while non-canonical type comprised the highest percentage of stroma with functional mitochondrial status. The mixed type and non-canonical type of glutamine-dependent metabolism were significantly associated with shorter overall survival than the other two types (P<0.001). To sum up, the mixed type and non-canonical type of glutamine-dependent metabolism in pancreatic cancer were more metabolic active, biologically aggressive, and had a relatively poorer prognosis than the other two types.

Next, we classified the tumors into following types according to the two categories of metabolism: glucose-dependent type, glutamine-dependent type, mixed type, and null type. Results above suggested that most tumors utilized both glucose- and glutamine-dependent metabolism to survive under shortage of nutrients and oxygen. We also found that highly glycolytic tumors were more dependent on glutamine-dependent metabolism and vice versa, which may be helpful for tumor growth and survival. Further, we divided the patients into groups according to the numbers of metabolic types and found that the overall survival was significantly longer in one-type group than two-type or three-type group. Moreover, two-type group show a relatively better prognosis than three-type group. Results above suggested that the increasing numbers of metabolism types inversely associated with survival outcome. The more subtypes and categories of metabolism pancreatic cancer utilizes, the worse outcome it is. It is reasonable that pancreatic cancers can reprogram metabolism and rely on two or more metabolism types and categories to adapt themselves to environmental pressures and support their rapid growth.

Indeed, various clinicopathological parameters, including tumor size, tumor location, tumor histological grade, lymphatic/vascular invasion, and resection margin status, have an impact on prognosis of patients with pancreatic cancer after tumor resection [31,32]. However, the relation between clinicopathological parameters and prognosis are obscure and hard to grasp. Therefore, some more adequate preoperative identification methods of patients with poor prognosis are desirable to aid appropriate clinical decision making. We demonstrated here that expression of metabolism-related proteins, status of glutamine-dependent metabolism, subtypes and categories of metabolism which pancreatic cancer utilizes may be good prognostic factors. By assessing the status of glucose-dependent and glutamine-dependent metabolism through endoscopic ultrasound-fine needle aspiration in a prospective manner, classification of pancreatic cancer patients may be achievable and more adequate clinical decisions will be made.

We acknowledge that our present study has following limitations. First, the study design is partly retrospective and the number of patients is not large enough. Second, though all of the patients receive the same surgery (pancreaticoduodenectomy) and received gemcitabine chemotherapy after surgery, the other postoperative treatment of these patients varied. But results above appeared to be not affected by those different postoperative treatments. Some results (i.e. the relation of lymphatic invasion and overall survival) in this study were inconsistent with previous data, which may be attributed to possible differences in ethnic incidence.

## Conclusion

Pancreatic cancer is heterogeneous in its metabolic phenotypes, and classification into various metabolic phenotypes may be helpful for more adequate clinical decision of therapy targeting metabolism and improving prognosis. To sum up, Warburg type, non-canonical type and mixed types of these two metabolisms consisted of metabolically active, biologically aggressive and poor prognosis tumors. Further, we identified for the first time that increasing numbers of the metabolism subtypes and categories robustly reflected major differences in survival outcome. The more subtypes and categories of metabolism pancreatic cancers employ, the worse outcome it is.

## Supporting Information

S1 File Supporting figures for the present study. Figure A in S1 File. Typical immunohistochemical labeling of positive control of various metabolism-related proteins. Figure B in S1 File. Kaplan-Meier survival curves for patients after surgery for pancreatic ductal adenocarcinoma demonstrating relationships of metabolism-related proteins with postoperative overall survival. Figure C in S1 File. Transcript levels of various genes including SLC2A1(solute carrier family 2 (facilitated glucose transporter), member 1), CAIX (carbonic anhydrase IX), BNIP3 (BCL2/adenovirus E1B 19kDa interacting protein 3), BECN1 (beclin 1, autophagy related), MAP1LC3A (microtubule-associated protein 1 light chain 3 alpha), SQSTM1 (sequestosome 1), GLUD1 (glutamate dehydrogenase 1), GOT1 (glutamic-oxaloacetic transaminase 1) were determined by quantitative qRT-PCR, results are means ± SD of 2 independent experiments done in triplicate. Three normal pancreas tissues were chosen as negative controls. *P<0.05, **P<0.01, ***P<0.005 versus controls.
(PDF)

S2 File Supporting tables for the present study, including antibody information, classification of tumor metabolic phenotypes, primers of metabolic genes and so on. (PDF)
