# Supplementary figures and images for: Metabolic Phenotypes in Pancreatic Cancer
Source: PLoS One. 2015 Feb 26;10(2):e0115153. doi: 10.1371/journal.pone.0115153 (PMC4342019; doi:10.1371/journal.pone.0115153)

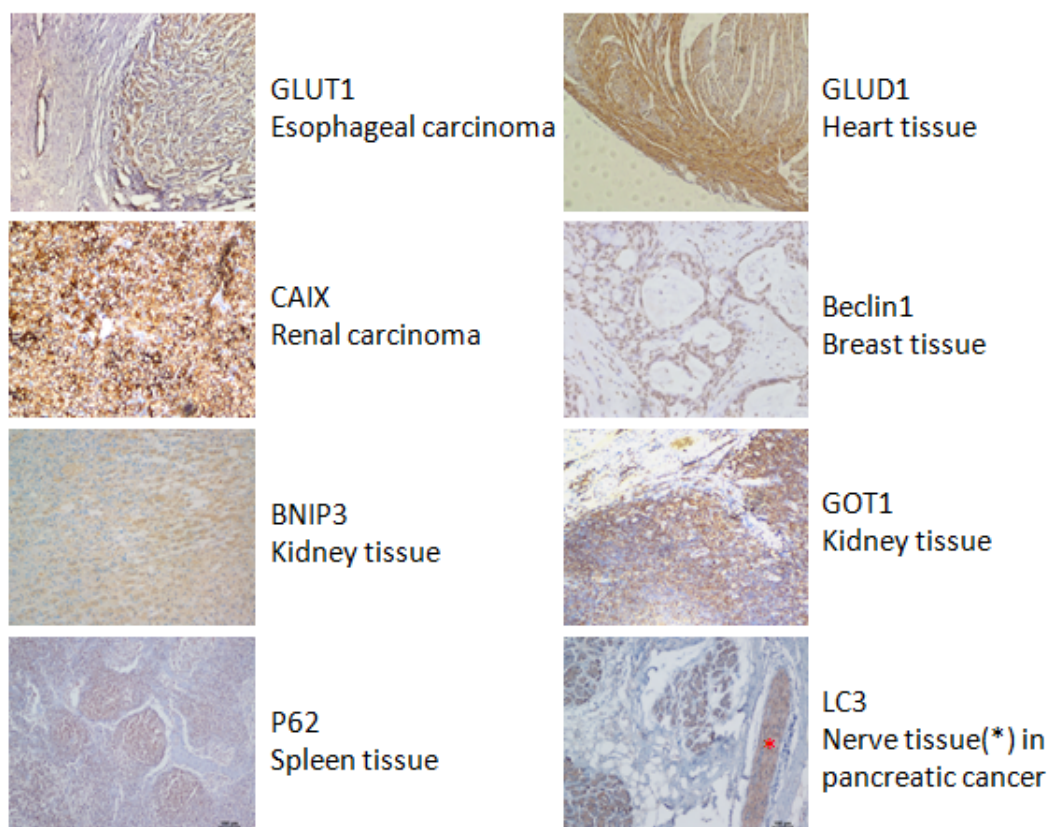

Figure A

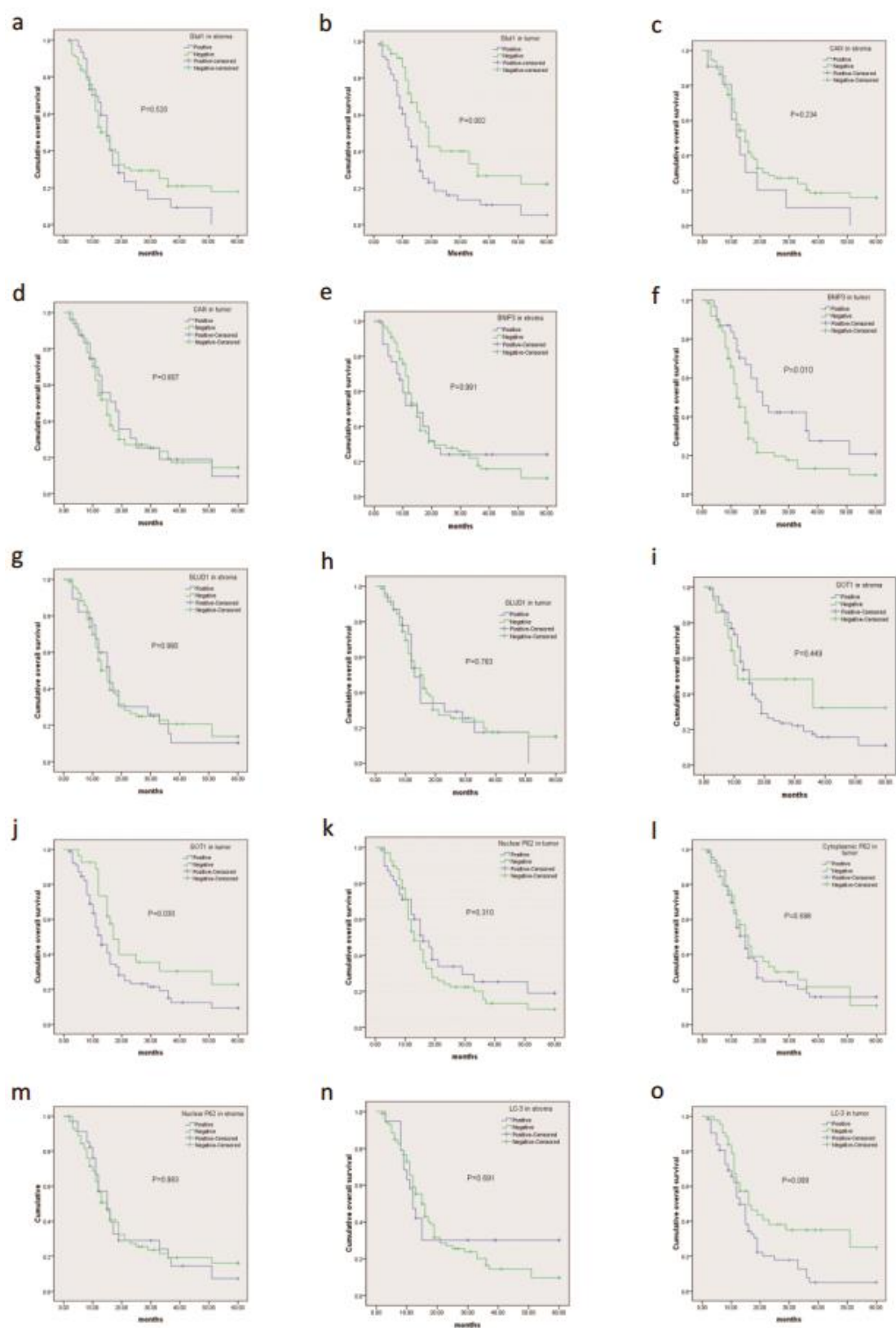

Figure B

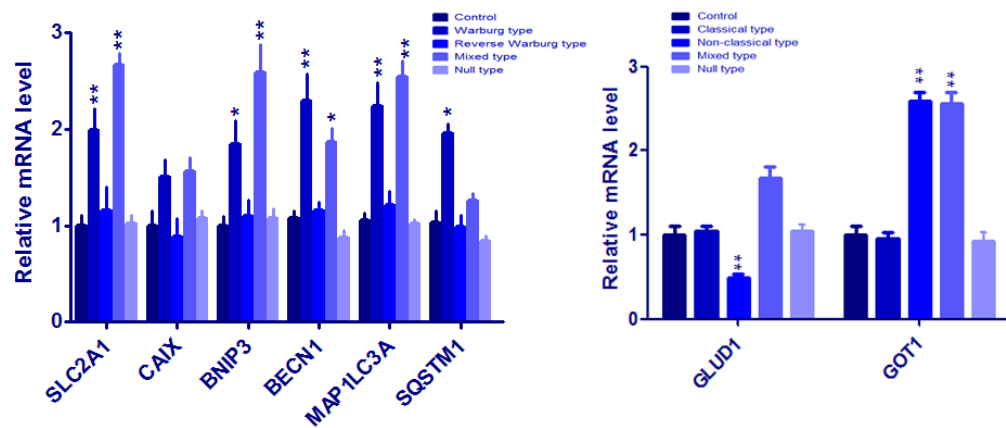

Figure C

Supplement: S1 File — Figure A in S1 File. Typical immunohistochemical labeling of positive control of various metabolism-related proteins. Figure B in S1 File. Kaplan-Meier survival curves for patients after surgery for pancreatic ductal adenocarcinoma demonstrating relationships of metabolism-related proteins with postoperative overall survival. Figure C in S1 File. Transcript levels of various genes including SLC2A1(solute carrier family 2 (facilitated glucose transporter), member 1), CAIX (carbonic anhydrase IX), BNIP3 (BCL2/adenovirus E1B 19kDa interacting protein 3), BECN1 (beclin 1, autophagy related), MAP1LC3A (microtubule-associated protein 1 light chain 3 alpha), SQSTM1 (sequestosome 1), GLUD1 (glutamate dehydrogenase 1), GOT1 (glutamic-oxaloacetic transaminase 1) were determined by quantitative qRT-PCR, results are means ± SD of 2 independent experiments done in triplicate. Three normal pancreas tissues were chosen as negative controls. *P<0.05, **P<0.01, ***P<0.005 versus controls. (PDF) [file pone.0115153.s001.pdf]
