# Supplementary material for: Metabolic Phenotypes in Pancreatic Cancer
Source: PLoS One. 2015 Feb 26;10(2):e0115153. doi: 10.1371/journal.pone.0115153 (PMC4342019; doi:10.1371/journal.pone.0115153)
Supplement: S2 File — (PDF) [file pone.0115153.s002.pdf]

Table 1. Source, clone and dilution of antibodies used in this study

Antibody	Clone	Dilution	Company
Glycolysis-related			
GLUT1	Monoclonal	1:250	Epitomics, California, USA
CAIX	Monoclonal	1:100	Epitomics, California, USA
Mitochondrial satus-related			
BNIP3	Monoclonal	1:50	Epitomics, California, USA
Glutamine metabolism			
GLUD1	Monoclonal	1:100	Epitomics, California, USA
GOT1	Monoclonal	1:100	Abgent, San Diego, USA
Autophagy-related			
Beclin-1	Monoclonal	1:150	Epitomics, California, USA
LC3	RB7481	1:100	Abgent, San Diego, USA
P62	Monoclonal	1:100	Abcam, Cambridge, UK

Table 2. Classification of tumor metabolic phenotypes according to the expression of metabolism-related proteins.

	Glucose-derived metabolism				Glutamine-derived metabolism	
	Warburg effect		Reverse Warburg effect		Classical	Non-classical
Location	Tumor	Stroma	Tumor	Stroma	Tumor	Tumor
Metabolism	Glycolysis	OXPHOS	OXPHOS	Glycolysis	GLUD1 dependant Glutaminolysis	GOT dependant Glutaminolysis
Expressions of Metabolism -related proteins	GLUT1+ and CAIX+; GLUT1- and CAIX+; GLUT1+ and CAIX-	GLUT1- and CAIX-	GLUT1- and CAIX-	GLUT1+ and CAIX+; GLUT1- and CAIX+; GLUT1+ and CAIX-	GLUD1+ and GOT1-	GLUD1- and GOT1+

Table 3. Pairwise comparisons of Kaplan-Meier survival curves for patients after surgery for pancreatic ductal adenocarcinoma demonstrating relationships of the glucose-dependent metabolism phenotypes with postoperative overall survival.

Glucose-derived metabolism	Warburg type		Reverse Warburg type		Mixed type		Null type	
	χ^2	Sig.	χ^2	Sig.	χ^2	Sig.	χ^2	Sig.
Warburg type			4.513	0.034	0.481	0.488	3.907	0.048
Reverse Warburg type	4.513	0.034			7.433	0.006	0.208	0.648
Mixed type	0.481	0.488	7.433	0.006			4.450	0.035
Null type	3.907	0.048	0.208	0.648	4.450	0.035		

Table 4. Pairwise comparisons of Kaplan-Meier survival curves for patients after surgery for pancreatic ductal adenocarcinoma demonstrating relationships of the glutamine-derived metabolism phenotypes with postoperative overall survival.

Glutamine-derived metabolism	Classical type		Non-classical type		Mixed type		Null type	
	χ^2	Sig.	χ^2	Sig.	χ^2	Sig.	χ^2	Sig.
Classical type			6.737	0.009	21.704	0.000	0.563	0.453
Non-classical type	6.737	0.009			16.803	0.000	4.814	0.028
Mixed type	21.704	0.000	16.803	0.000			15.425	0.000
Null type	0.563	0.453	4.814	0.028	15.425	0.000		

Table 5. Pairwise comparisons of Kaplan-Meier survival curves for patients after surgery for pancreatic ductal adenocarcinoma demonstrating relationships of the two subtypes of metabolisms with postoperative overall survival.

	Glutamine-dependent type		Glucose-dependent type		Mixed type		Null type	
Metabolism types	χ^2	Sig.	χ^2	Sig.	χ^2	Sig.	χ^2	Sig.
Glutamine-dependent type			1.172	0.279	0.369	0.544	4.402	0.036
Glucose-dependent type	1.172	0.279			1.160	0.281	5.246	0.022
Mixed type	0.369	0.544					0.512	0.474
Null type	4.402	0.036	5.246	0.022	0.512	0.474		

Table 6. Pairwise comparisons of Kaplan-Meier survival curves for patients after surgery for pancreatic ductal adenocarcinoma demonstrating relationships of the metabolism phenotypes with postoperative overall survival.

Combination of metabolism subtypes	Zero type		One type		Two type		Three type		Four type	
	$\chi 2$	Sig.	$\chi 2$	Sig.	$\chi 2$	Sig.	$\chi 2$	Sig.	$\chi 2$	Sig.
Zero type			2.396	0.122	4.380	0.036	5.756	0.016	3.000	0.083
One type	2.396	0.122			0.742	0.039	5.886	0.005	15.000	0.000
Two type	4.380	0.036	0.742	0.039			5.089	0.024	11.764	0.001
Three type	5.756	0.016	5.886	0.005	5.089	0.024			8.5000	0.004
Four type	3.000	0.083	15.000	0.000	11.764	0.001	8.5000	0.004		

Table 7. The primers of metabolism related genes.

**Accession No.**	**Gene**	**Primer sequence （5'to3'）**		**Products（bp）**	**Tm（℃）**
AK292791	GLUT1	Forwards	GCGGTTTTCTATTACTCCACAAGC	122	62.0
		Reverse	TCCACCACGAACAGCGACAC		
AI023541	CAIX	Forwards	GACCTTGTTGGAATGGCTCTT	160	60.2
		Reverse	TGGATTCAGGTGCAAATGCAA		
AF002697	BNIP3	Forwards	TTGGATGCACAACATGAATCAGG	140	61.0
		Reverse	TCTTCTGACTGAGAGCTATGGTC		
AF077301	BECN1	Forwards	GGTGTCTCTCGCAGATTCATC	121	61.4
		Reverse	TCAGTCTTCGGCTGAGGTTCT		
AF276658	MAP1LC3A	Forwards	AACATGAGCGAGTTGGTCAAG	127	58
		Reverse	GCTCGTAGATGTCCGCGAT		
AK098077	SQSTM1	Forwards	GACTACGACTTGTGTAGCGTC	139	61.1
		Reverse	AGTGTCCGTGTTTCACCTTCC		
AK094782	GLUD1	Forwards	CTGTGGTCGATGTACCGTTTG	109	61.5
		Reverse	AGCTCCATAGTGAACCTCCGT		
AF052153	GOT1	Forwards	CCACCCTTTCAGTGTTCATGG	131	61.2
		Reverse	GCCTTCATTTGTCATGCAGACT		
